# Catatonia in patients with dementia: a case report

**DOI:** 10.11604/pamj.2019.33.117.18629

**Published:** 2019-06-14

**Authors:** Samira Younes, Sabria Khouadja, Samia Younes, Lazhar Zarrouk

**Affiliations:** 1 Department of Psychiatry, University Hospital of Mahdia, Mahdia, Tunisia; 2 Department of Neurology, University Hospital of Mahdia, Mahdia, Tunisia

**Keywords:** Catatonia, dementia, lorazepam

## Abstract

Catatonia occurring as part of a clinical picture of dementia has been reported with almost all types of dementia. It remains under-diagnosed in older adults and those with dementia. We review a case of a young patient admitted in our psychiatric department for catatonia and after efficient treatment with Lorazepam, assessment revealed a dementia. Catatonia is a severe neuropsychiatric syndrome with an excellent prognosis if recognized and treated without delay.

## Introduction

In 1874, Karl Kahlbaum described catatonia in patients who suffered from severe psychotic, mood and medical conditions [1]. Kraepelin and Bleuler, however, redefined catatonia as a subtype of dementia praecox [2] and schizophrenia [3]. In a Dutch study, clinicians could identify catatonia in only 2% of 139 inpatients, but the research team was able to identify catatonia in 18% [4], indicating that the diagnosis of catatonia is often missed. Catatonia is treatable once it is diagnosed [5]. The present study aims to describe the characteristics of catatonia in patients with dementia and the efficiency of early management.

## Patient and observation

**Figure 1:**
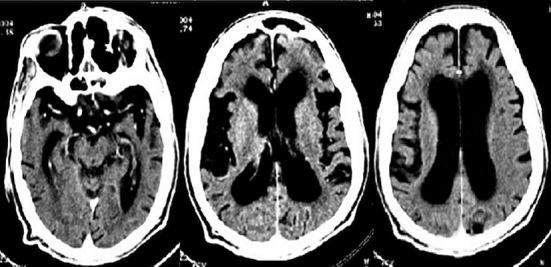
Brain scan showing a cortical and sub-cortical atrophy predominant in the bilateral fronto-temporo-parietal region associated with ventricular system expansion

A 49-year-old male with a psychiatric history of an acute psychotic episode at age of 35 years treated with classic antipsychotic drug (Haloperidol). Three years later, the patient was admitted to a psychiatric ward for behavioral disorders with delirium of erotomania and persecution through an interpretive and intuitive mechanism with confusion. The brain scan showed cortical and subcortical atrophy. Clinical and biological exams were without abnormalities. A diagnostic presumption of Alzheimer's disease was made and the patient was treated with high-doses of antipsychotic drugs with vitamin therapy and vasodilator treatment. He has a follow-up at the outpatient psychiatric consults. His family described a cognitive decline and variation in psychotic symptoms with a progressive limitation of his self-reliance and he would have ceased all professional activities with social withdrawal. Currently, ten years later, he was hospitalized in a stuporous state associated with food refusal, sustained posture and worsening of his overall situation. At the mental assessment, the patient was motionless, mute and rigid with frozen facial expression and gaze stare. Negativity and opposition were obvious against any solicitation. It was hard to make any contact because of nodding or blinking answers. Moreover, the physical examination has shown a worsening of the overall state of health with marked weight loss and walking difficulties. After symptomatic treatment of catatonia with benzodiazepine (Lorazepam) and vitamin therapy, the assessment revealed an aphaso-apraxo-agnotic syndrome associated with memory dysfunctions such as anterograde and retrograde amnesia with false recognition and executive dysfunction as well as limitations in intellectual abilities (acalculia, lack of abstraction). A brain scan revealed cortical and subcortical atrophy predominant in the bilateral fronto-temporo-parietal region associated with ventricular system expansion (Figure 1). Biological tests and serologies were normal. The diagnosis of Alzheimer's disease was made. Following atypical antipsychotic treatment combined with benzodiazepine, there was relative stabilization with release of inhibition, improvement of contact and recovery of appetite. However, the patient remained reliant on a caregiver and there was a significant cognitive decline with a loss of memory, executive and instrumental functions.

## Discussion

Catatonia occurring as part of clinical picture of dementia has been reported with almost all types of dementia, i.e. Alzheimer's dementia [6, 7], dementia of Lewy body [8-11], fronto-temporal dementia (FTD) [12-14], and dementia due to any general medical condition (AIDS, stroke, etc.) [15]. Catatonia is a treatable neuropsychiatric disorder characterized by motor, behavioral, and autonomic abnormalities [16], which remains under-diagnosed in older adults and those with dementia [15, 17]. In this group, its frequency is unclear, etiology tends to be multifactorial [15, 17], and there is a greater risk of complications if undiagnosed or untreated [17]. Cuevas-Esteban *et al*. [18] used the Bush-Francis Catatonia Rating Scale (BFCSI) [19] to assess catatonia in 106 patients who were admitted to an acute geriatric psychiatry ward. Catatonia was highly prevalent (n=42; 39.6%) and seventeen patients (16%) were diagnosed as suffering from dementia. Using the same methodology, Puja *et al*. [20], have found that the rate of catatonia in patients with dementia was 42.8% (6 out of 14). Etiology of catatonia appeared to be multifactorial, including structural brain disease (degenerative and vascular) due to dementia, and subsequent accrual of cognitive impairment; the presence of one or more chronic vascular risk factors; the occurrence of delirium secondary to a urine infection and a depressive episode; and exposure to antipsychotic drugs before admission in four out of six patients. There were no major complications in any patient. Five out of six of these patients received treatment for catatonia with lorazepam, all achieving complete remission and there were no recurrences. The early detection and treatment of catatonia in dementia can lead to significant symptomatic improvement, and this may help prevent potentially serious complications [15, 17]. About 70% of catatonic patients respond to lorazepam alone, regardless of the cause of catatonia [21]. Electroconvulsive therapy (ECT) is another effective treatment that works even when benzodiazepines fail to give the desired response [22]. Jaimes-Albornoz *et al*. [13] reported two patients with fronto-temporal dementia that present a catatonic state. First case was a 65 year old female who was hospitalized after losing weight due to her active refusal to eat. Once stabilized physically was derived to psycho-geriatric ward where a catatonic syndrome was observed and she was treated within one week with lorazépam 2.5mg/day and zolpidem 10mg/day. Perseverative behavior and mannerisms was maintained. Second case was a 67 year old male who was admitted to psycho-geriatric ward because he had aggressivity and negativism. Catatonia was diagnosed and he was treated with lorazepam 10mg/day and valproic acid 900mg/day. Catatonic symptoms disappeared a month later. Negativism and impulsiveness persisted slightly.

## Conclusion

Catatonia is a severe neuropsychiatric syndrome with an excellent prognosis if recognized and treated without delay. Catatonia in dementia is rare but not uncommon. Clinicians should be aware of catatonia as a diagnostic possibility in patients with dementia and look for it proactively.

## Competing interests

The authors declare no competing interests.

## References

[1] Kahlbaum K (1873). Die Katatonie oder das Spannungs-Irresein.

[2] Kraepelin E (1919). Dementia Praecox and Paraphrenia.

[3] Bleuler E (2010). Dementia Praecox or the Group of Schizophrenias. Vertex.

[4] van der Heijden FM, Tuinier S, Arts NJ, Hoogendoorn ML, Kahn RS, Verhoeven WM (2005). Catatonia: disappeared or under-diagnosed. Psychopathology.

[5] Ungvari GS, Leung CM, Wong MK, Lau J (1994). Benzodiazepines in the treatment of catatonic syndrome. Acta Psychiatr Scand.

[6] Alisky JM (2004). Is the immobility of advanced dementia a form of lorazepam-responsive catatonia. Am J Alzheimers Dis Other Demen.

[7] Kendurkar A (2008). Catatonia in an Alzheimer's dementia patient. Psychogeriatrics.

[8] Fekete R (2013). Renal failure in dementia with lewy bodies presenting as catatonia. Case Rep Neurol.

[9] Lakshmana R, Sundram S, Cairns F (2006). Dementia with Lewy bodies (DLB) presenting with catatonic symptoms. Psychogeriatrics.

[10] Morita S, Miwa H, Kondo T (2004). A patient with probable dementia with Lewy bodies, who showed catatonia induced by donepezil: a case report. No To Shinkei.

[11] Ueda S, Koyama K, Kocha H, Okubo Y (2011). Dementia with Lewy bodies (DLB) accompanied by symptoms of late catatonia: what to consider and how to treat. Seishin Shinkeigaku Zasshi.

[12] Utumi Y, Iseki E, Arai H (2013). Three patients with mood disorders showing catatonia and frontotemporal lobes atrophy. Psychogeriatrics.

[13] Jaimes-Albornoz W, Ballesteros-Prado A, Serra-Mestres J (2015). Catatonia in Patients with Frontotemporal Dementia. Eur Psychiatry.

[14] Sheikhi L, Li Y, Jimenez XF (2015). A case of familial frontotemporal dementia presenting with malignant catatonia. Neurol Clin Pract.

[15] Takata T, Takaoka K, Fujigaki M (2005). Catatonia in the elderly. Int J Psychiatry Clin Pract.

[16] Fink M (2013). Rediscovering catatonia: the biography of a treatable syndrome. Acta Psychiatr Scand Suppl.

[17] Jaimes-Albornoz W, Serra-Mestres J (2013). Prevalence and clinical correlations of catatonia in older adults referred to a liaison psychiatry service in a general hospital. Gen Hosp Psychiatry.

[18] Cuevas-Esteban J, Iglesias-González M, Rubio-Valera M, Serra-Mestres J, Serrano-Blanco A, Baladon L (2017). Prevalence and characteristics of catatonia on admission to an acute geriatric psychiatry ward. Prog Neuropsychopharmacol Biol Psychiatry.

[19] Bush G, Fink M, Petrides G, Dowling F, Francis A (1996). Catatonia I rating scale and standardized examination. Acta Psychiatr Scand.

[20] Sharma P, Sawhney I, Jaimes-Albornoz W, Serra-Mestres J (2017). Catatonia in patients with dementia admitted to a geriatric psychiatry ward. J Neurosci Rural Pract.

[21] Fink M, Shorter E, Taylor MA (2010). Catatonia is not schizophrenia: Kraepelin's error and the need to recognize catatonia as an independent syndrome in medical nomenclature. Schizophr Bull.

[22] Bush G, Fink M, Petrides G, Dowling F, Francis A (1996). Catatonia II Treatment with lorazepam and electroconvulsive therapy. Acta Psychiatr Scand.

